# Comparative study of four different types of intraperitoneal mesh prostheses in rats^1^

**DOI:** 10.1590/s0102-865020190070000003

**Published:** 2019-09-12

**Authors:** Rogério Aoki Fuziy, Ricardo Artigiani, Elesiario Marques Caetano, Ana Karina Soares Alves, Gaspar Jesus Lopes, Marcelo Moura Linhares

**Affiliations:** I Fellow Master degree, Postgraduate Program in Interdisciplinary Surgical Sciences, Division of Surgical Gastroenterology, Department of Surgery, Universidade Federal de São Paulo (UNIFESP), Brazil. Design of the study; acquisition, analysis and interpretation of data; manuscript preparation; critical revision; final approval.; II PhD, Division of Surgical Gastroenterology, Department of Surgery, UNIFESP, Sao Paulo-SP, Brazil. Design of the study; acquisition, analysis and interpretation of data; manuscript preparation; critical revision; final approval.; III MD, Division of Surgical Gastroenterology, Department of Surgery, UNIFESP, Sao Paulo-SP, Brazil. Acquisition and interpretation of data, critical revision, final approval.; IV PhD, Chairman, Division of Surgical Gastroenterology, Department of Surgery, UNIFESP, Sao Paulo-SP, Brazil. Acquisition and interpretation of data, critical revision, final approval.; V PhD, Full Professor, Division of Surgical Gastroenterology, Department of Surgery, UNIFESP, Sao Paulo-SP, Brazil. Design of the study; acquisition, analysis and interpretation of data; critical revision; final approval.

**Keywords:** Tissue Adhesions, Surgical Mesh, Cyclooxygenase 2, Rats

## Abstract

**Purpose:**

To compare four types of mesh regarding visceral adhesions, inflammatory response and incorporation.

**Methods:**

Sixty Wistar rats were divided into four groups, with different meshes implanted intraperitoneally: polytetrafluoroethylene (ePTFE group); polypropylene with polydioxanone and oxidized cellulose (PCD); polypropylene (PM) and polypropylene with silicone (PMS). The variables analyzed were: area covered by adhesions, incorporation of the mesh and inflammatory reaction (evaluated histologically and by COX2 immunochemistry).

**Results:**

The PMS group had the lowest adhesion area (63.1%) and grade 1 adhesions. The ePTFE and PM groups presented almost the total area of their surface covered by adherences (99.8% and 97.7% respectively) The group ePTFE had the highest percentage of area without incorporation (42%; p <0.001) with no difference between the other meshes. The PMS group had the best incorporation rate. And the histological analysis revealed that the inflammation scores were significantly different.

**Conclusions:**

The PM mesh had higher density of adherences, larger area of adherences, adherences to organs and percentage of incorporation. ePTFE had the higher area of adherences and lower incorporation. The PMS mesh performed best in the inflammation score, had a higher incorporation and lower area of adherences, and it was considered the best type of mesh.

## Introduction

Incisional hernias are one of the most frequent postoperative complications^1^ Obesity, advanced age, diabetes, pulmonary diseases, malnutrition and multiple surgeries at the same site are predisposing factors. Several hernia correction methods have been proposed; yet none is considered the gold standard in the repair of incisional hernias, and the treatment of obese patients, with multiple hernia lesions or abdominal wall loss is even more complex^2^.

The repair of incisional hernias using simple closing techniques or the Mayo procedure is recommended to fix small defects with less than 5 cm, but they may have a recurrence rate above 50%^3^ ^,^ ^4^. For large incisional hernias, techniques using mesh present lower recurrence and hazard rates than techniques without mesh reinforcement, according to a recent metanalysis^1^. The use of mesh has indeed become standardized in incisional hernias, allowing the reduction of the recurrence rate to 5.2-24%^5^ ^,^ ^6^. Surgical treatment of large incisional hernias aims to repair the defect and to normalize the containment function of the abdominal wall, and synthetic prostheses reinforce the suture and replace missing tissue^7^. However, despite better results with synthetic prostheses than with simple closure, they can cause severe complications, depending on the positioning in which they are fixated on the abdominal wall^8^.

Three sites are suitable for the positioning of the mesh: on the aponeurosis, pre-peritoneally and intraperitoneally. Positioning on the aponeurosis is the most commonly used method, but it is associated with higher recurrence rate and higher incidence of postoperative complications, such as wound infection, hematoma and seroma formation^8^. In the pre-peritoneal position, the mesh is protected from the abdominal contents through the posterior fascia of the rectus abdominis muscle and peritoneum, or only the peritoneum, if it is positioned posteriorly to the fascia. This technique is described as leading to a lower rate of adhesion formation and fewer postoperative complications^9^.

Intraperitoneal position was used in the past, with interposition of the omentum on the abdominal viscera. Contact of the mesh with the intestines can occur when the peritoneum closure is not possible, and the surgeon tries a tension-free repair. The use of intraperitoneal mesh was associated with firm adhesions, intestinal lesions, migration and erosion of the mesh by the adjacent organs with formation of enterocutaneous fistula^10^ ^,^ ^11^, although the association of the fistula with the positioning of intraperitoneal polypropylene mesh has not been observed in all studies^12^.

To avoid this serious complication, the industry developed mesh screens coated with absorbable material, which act as a temporary barrier on the polypropylene framework and the abdominal viscera, such as the polypropylene mesh coated with hyaluronic acid, or nonabsorbable material, such as expanded polytetrafluoroethylene (ePTFE) and polypropylene coated with silicone^13^ ^–^ ^16^. The polypropylene material can also be embedded with anti-adherent solutions of hyaluronic acid or icodextrin or coated with methylcellulose, hyaluronic acid or titanium; the polyester mesh may be embedded with collagen^17^. The possibility of adding an absorbable non-adherent solution made with polyethylene glycol hydrogel has been investigated too^18^.

Each type of mesh is different in porosity and thickness, as well as density, which potentially influences the incorporation into the scar tissue and the foreign body reaction or biocompatibility^19^ ^,^ ^20^, as well as adhesions. However, although the studies have compared, usually two by two, different mesh materials for intra peritoneal repair, it is still necessary to evaluate the adherence of prosthetic mesh to abdominal viscera as well as its incorporation to the abdominal wall and a local inflammatory reaction. Therefore, the objective of the present study is to compare four different products regarding visceral adherences and tissue inflammatory reaction: a polypropylene mesh, a polypropylene mesh with encapsulated polydioxanone and coated with oxidized cellulose, a polypropylene mesh coated with silicone and a polytetrafluoroethylene expanded mesh. The hypothesis was that incorporation, adhesions and local inflammatory reaction would be different between the mesh materials.

## Methods

This research protocol was approved by the institutional ethics committee (protocol CEP 1292/11), as it fully adheres to all national and institutional guidelines for the care and use of the animals used in experimental researches, and also the current national laws.

### Study design, experimental animals and housing and husbandry

This is an experimental surgical study with Wistar rats, comparing four different mesh support products for hernia repair. The experimental procedures were performed always in the same laboratory, a light and temperature-controlled room. All the animals used in this study had been bred in the same university *bioterium,* and were healthy before the experiment. All rats were male and weighted around 250 grams.

A convenience sample of 60 rats was used. Rats were identified with numbers and were maintained in groups of 5 animals per cage. They were observed during the study period for autophagia, mutilating behavior, infections and body movements. Rats were fed and hydrated ad libitum. The size of the cages was 30 cm x 40 cm x 20 cm. After identification with numbers, the animals were randomly allocated in four groups of 15 animals using electronic random allocation sequence generation.

Four different compositions of mesh products were used in each group, as follows:

- ePTFE group: polytetrafluoroethylene expanded mesh (Gore-Tex Dual Mesh; Gore-tex United States);
- PCD group: polypropylene mesh encapsulated with polydioxanone and coated with oxidized cellulose (Proceed, Ethicon, United States);
- PM group: polypropylene mesh (Prolene, Ethicon, United States);
- PMS group: polypropylene mesh coated with silicone (Implants, Microval, France).

### Surgical procedures

Rats were fasted for 8 hours prior to surgery. Anesthesia was made with ketamine hydrochloride in combination with xylazine (10% and 2%), by intraperitoneal injection, at a dose of 0.1 ml of solution per 100g of body weight. Trichotomy of the anterior abdominal wall, with electrical appliance, was followed by antisepsis with iodopovidine topical solution. Sterile techniques were used during all surgeries.

**Figure 1:**
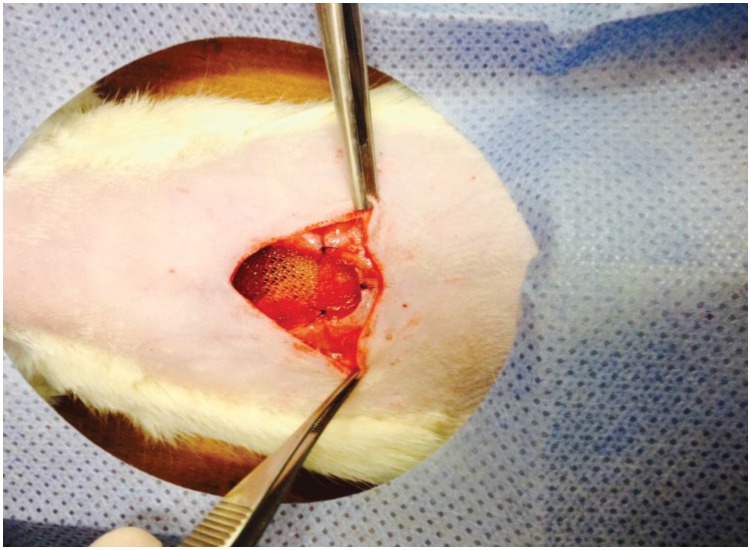
Experimental incisional hernia repair: final aspect of the mesh fixation, in contact with viscera.

**Figure 2:**
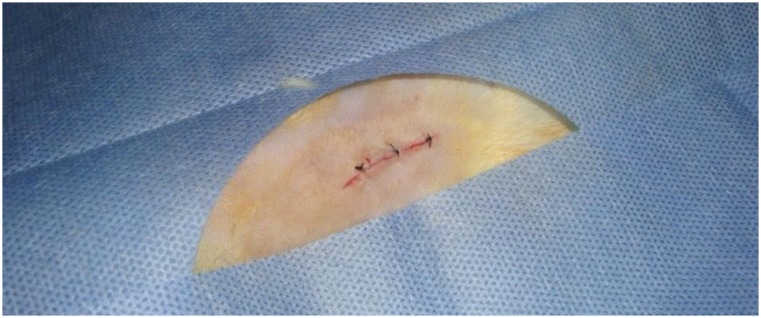
Skin suture with Vicryl 4-0.

The surgical technique model used in this study was the one proposed by Alponat *et al*.^13^ and Hooker *et al.* ^14^. A medial incision of 4 cm allowed the harvest of two skin patches that were separated from the abdominal wall, followed by the opening of the abdominal cavity through a longitudinal incision to the peritoneum, promoting a gap of 1.5 × 2.5 cm. All mesh products were cut into 3.5 cm × 2.5 cm square patches (area pf 8.75 cm^2^). In all animals the meshes were positioned intra peritoneal and in contact with intra-abdominal viscera, with six separate sutures of polypropylene 5-0, three sutures on each side. Skin was sutured with separate points of Vicryl 4-0 (Figs. 1 and 2).

### Necropsy and macroscopic evaluation

**Figure 3:**
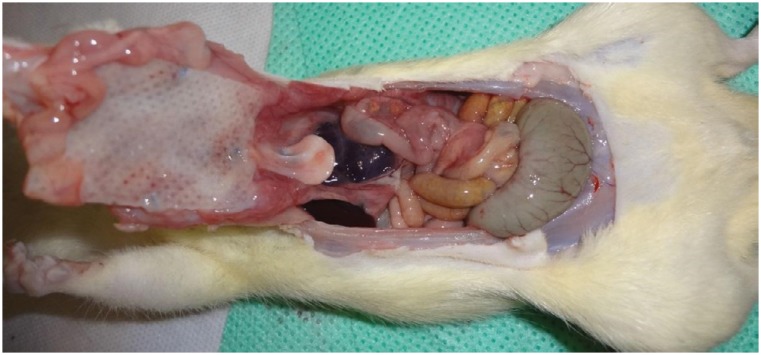
U-shaped incision in the abdominal cavity of the Wistar rat, after euthanasia.

**Figure 4:**
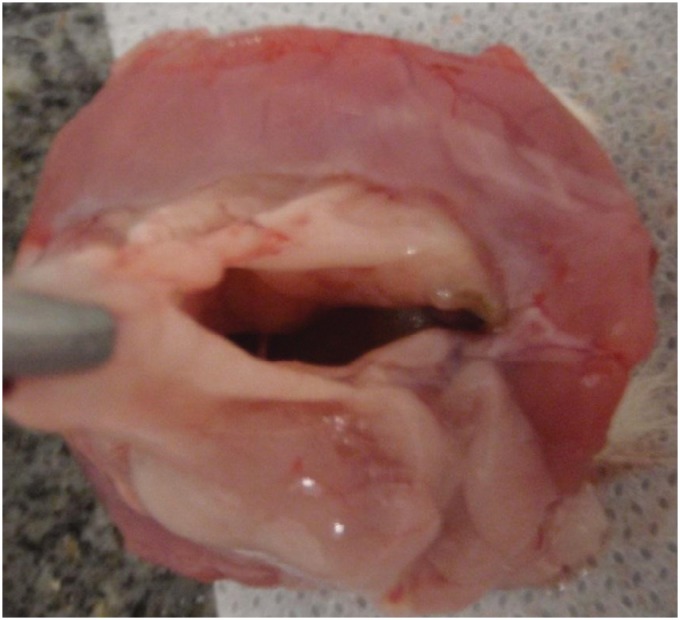
Purulent secretion in the wound.

**Figure 5:**
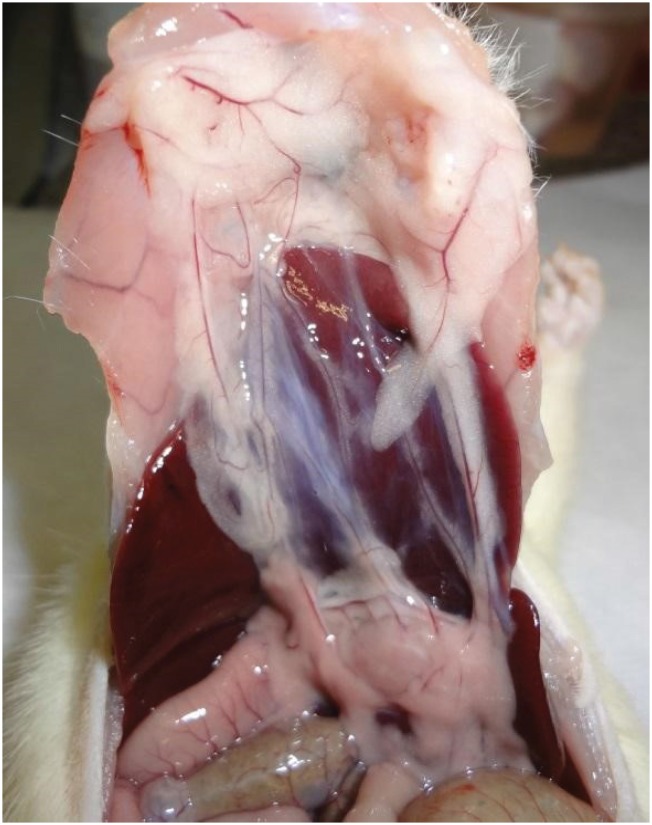
Adherence of abdominal viscera to the mesh.

Euthanasia was performed in the seventh postoperative day, followed by necropsy for histological evaluation. Rats were euthanized using 0.5 ml of ketamin and 0.5 ml of xilazin intraperitoneally. After euthanasia, the abdominal cavity of each rat was open in a U-shaped incision (Fig. 3) that contained the lateral and inferior regions of the mesh. Cases of infection were identified when there was pus in the wound or on the mesh (Fig. 4). In the macroscopic evaluation, the pathologist evaluated if there were adherences of the mesh to abdominal organs and incorporation of the mesh to the abdominal wall (Fig. 5).

The mesh was cut together with tissues adhered to it and the roots of visceral organs close to it. For the evaluation of the density of adhesions, the mesh surface was divided in six larger fields, and each of these six sections subsequently subdivided in other six fields, and for each field, the percentage of the surface covered by adhesions was calculated. The adhesions density was then classified as follows^15^ ^–^ ^17^:

Grade zero: no adherence;

Grade 1. Filmy adhesions, easy to separate by blunt dissection;

Grade 2, mild, but stronger adhesions, blunt dissection possible with partly sharp dissection;

Grade 3, moderate or strong adhesions, lysis possible, but with sharp dissection only;

Grade 4, very strong or severe adhesions, lysis is possible but with sharp dissection only; organ strongly attached can be damaged by dissection.

**Figure 6:**
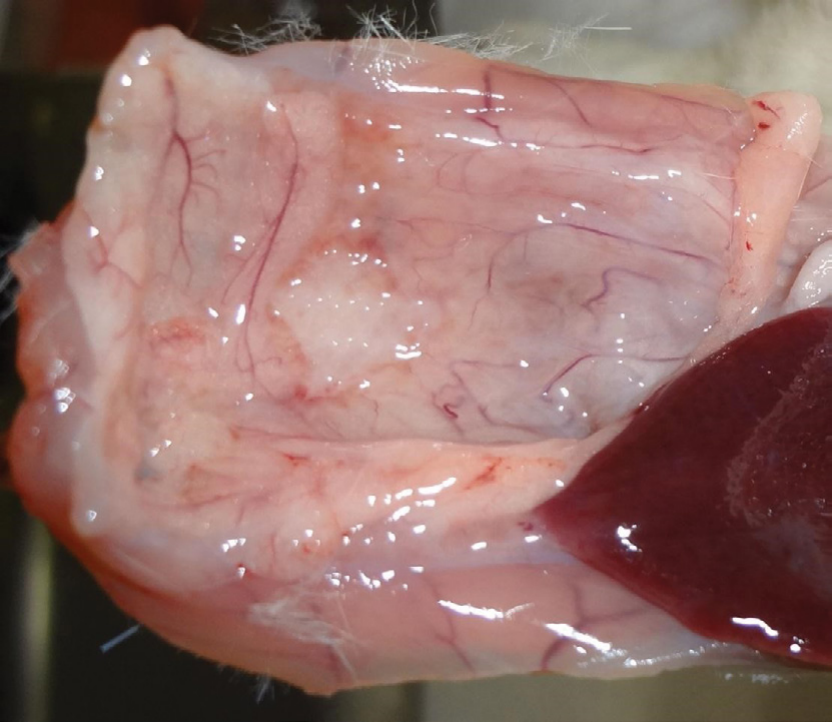
Area to be considered in the incorporation evaluation.

The incorporation area of the mesh to the omentum was measured by dividing the mesh in 10 sections and calculating the percentage of sections with incorporation (Fig. 6).

After these macroscopic evaluations, the material was fixed in buffered formalin (10%) and sent for histological analysis.

### Histological and immunohistochemistry evaluation

**Figure 7:**
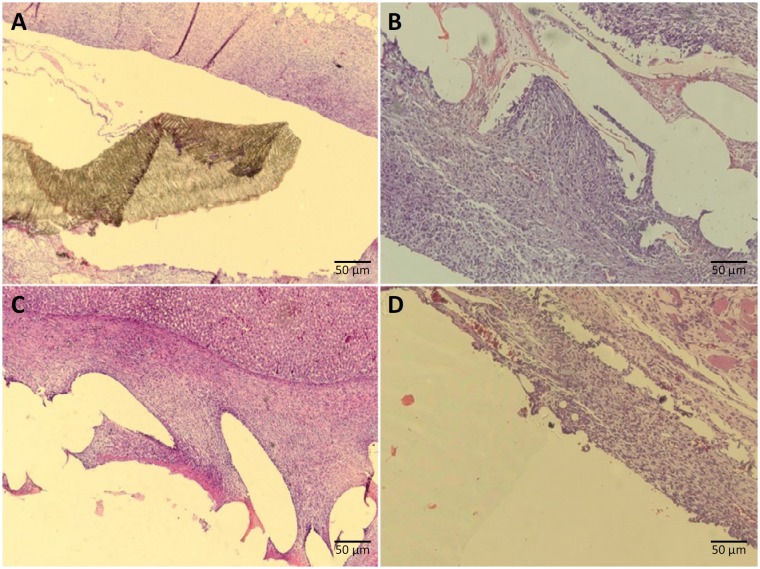
Hematoxylin-eosin stained slides of the transition between the mesh and the host tissue: **A.** ePTFE group: polytetrafluoroethylene expanded mesh; **B.** PCD group: polypropylene mesh encapsulated with polydioxanone and coated with oxidized cellulose; **C.** PM group: polypropylene mesh, and **D.** PMS group: polypropylene mesh coated with silicone.

After fixation, the tissue samples were treated with xylene and paraffin embedding, and were subjected to microtomy and hematoxylin-eosin (HE; Fig. 7) and Masson trichrome staining. One single experienced pathologist from the university team made all histological and immunohistochemistry evaluations. This pathologist was blind to animal allocation. Initially, using optical microscopy with increase of x200, the field to be evaluated was chosen at the transition between the mesh and the host tissue. Histological evaluation was then performed with magnification of x400.

For the inflammatory reaction evaluation, we used the scale proposed by Harrell *et al.* ^18^, with scores describing: A) the layers of cells in the periphery of the granulomas, with scores varying according to the number of layers; B) the inflammatory reaction in host tissue; C) the inflammatory response on the mesh surface; and D) the tissue maturation, with scores from 1 to 4. Two complementary evaluation items of the inflammatory reaction were also added (also with score values from 1 to 4), a classification proposed by Pereira-Lucena *et al*.^19^. This intends to evaluate the presence of giant cells and inflammatory invasion of muscles adjacent to the mesh.

**Figure 8:**
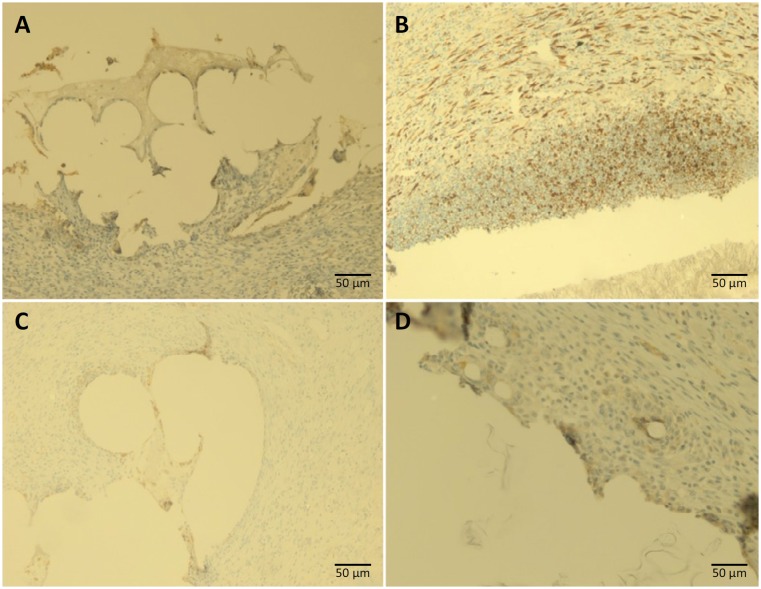
COX-2 evaluation by immunohistochemistry: **A.** ePTFE group: polytetrafluoroethylene expanded mesh; **B.** PCD group: polypropylene mesh encapsulated with polydioxanone and coated with oxidized cellulose; **C.** PM group: polypropylene mesh, and **D.** PMS group: polypropylene mesh coated with silicone.

The paraffin embedded blocks were used to prepare slides for COX2 evaluation (polyclonal Spring antibodies) by immunohistochemistry (Fig. 8), with the following steps:

- Antibody identification;
- Antigenic recovery;
- Blocking of endogenous peroxidase;
- Incubation with the primary antibody;
- Incubation with the one-step polymer (LSAB kit, Dako, K0640);
- Revelation and counterstain (DAB kit, Dako);
- Dehydration and assembly.

For objective data analysis, we used the Kim *et al.* ^20^ scale, with expression scores based on the percentage of stained cells and the intensity of the reaction, as shown in Table 1. This analysis was performed both in the peritoneal and the non-peritoneal sides of the mesh, because the mesh products can have different surfaces in different sides.

**Table 1:** Criteria for immunohistochemistry COX2 evaluation: amount of stained cells and intensity of reaction.

Percentage of stained cells		Points
	Up to 25%	1
	26% to 50%	2
	51% to 100%	3
**Intensity of reaction**		
	Mild	1
	Moderate	2
	Strong	3

### Statistical analysis

The Shapiro-Wilk test was used to test normality of quantitative variables. The chi-square and the Fisher's exact tests were used to verify associations between categorical variables and the four mesh types. For quantitative variables, the Kruskal-Wallis test was used, followed by paired Mann-Whitney test to better discriminate the statistical significance between two types of mesh.

The level of statistical significance was set at 0.05. Analyses were performed using the SPSS statistical package (version 18.0).

## Results

Two rats died during the experiments, one in the PMS group and the other in the PM group. Both deaths occurred in the immediate postoperative period and followed the increase of anesthetic doses for surgery. The study thus began with 14 animals in the PM and PMS groups and 15 in the PCD and ePTFE groups. However, it was not possible to perform immunohistochemistry analysis in all rats due to problems with the slides staining. COX-2 evaluation was performed in 14 animals in the PM and PCD groups, 13 animals in the PMS group and 12 animals in the ePTFE group.

The density of adhesions of the different mesh products is shown in Tables 2 and 3, while Table 4 shows the areas of lack of incorporation, with significant difference between groups. The results show that the incorporation area was the worst with the ePTFE group. Hematoma or seroma was present in all rats of the PTFE group, with a significant difference between groups (Table 5).

**Table 2:** Adherence scores per type of mesh.

Adherence score	ePTFE	PCD	PM	PMS	Total	Chi-squared	p	Fisher p
Grade 1	0 (0%)	0 (0%)	0 (0%)	13 (92.9%)	**13 (22.4%)**			
Grade 2	13 86.7%)	15 (100%)	1 (7.1%)	1 (7.1%)	**30 (51.7%)**	97.827	< 0.001	< 0.001
Grade 3	2 (13.3%)	0 (0%)	4 (28.6%)	0 (0%)	**6 (10.3%)**			
Grade 4	0 (0%)	0 (0%)	9 (64.3%)	0 (0%)	**9 (15.50%)**			
Total	15 (100%)	15 (100%)	14 (100%)	14 (100%)	**58 (100%)**			

PTFE: polytetrafluoroethylene expanded; PCD: polypropylene encapsulated with polydioxanone and coated with oxidized cellulose; PM: polypropylene mesh; PMS: polypropylene mesh coated with silicone.

**Table 3:** Estimated area of adherence per type of mesh.

Estimated % ofadherencearea	PTFE	PCD	PM	PMS
N	15	15	14	14
Mean	99.87%	83.20%	97.71%	63.14%
SD	0.52%	21.75%	5.81%	31.11%
Median	100.00%	92.00%	100.00%	70.00%
KW *x* ^2^		**25.816**		
*p-value*		**< 0.001**		
Mann-Whitney test (*p-value*)				
PTFE *vs*. PCD		0.002		
PTFE *vs*. PM		0.715		
PTFE *vs*. PMS		< 0.001		
PCD *vs*. PM		0.016		
PCD *vs.* PMS		0.07		
PM *vs.* PMS		0.001		

SD = standard deviation; PTFE: polytetrafluoroethylene expanded; PCD: polypropylene encapsulated with polydioxanone and coated with oxidized cellulose; PM: polypropylene mesh; PMS: polypropylene mesh coated with silicone.

**Table 4:** Not incorporated area of each type of mesh.

% notincoporatedarea	PTFE	PCD	PM	PMS
N	15	15	14	14
Mena	42.13%	15.47%	9.43%	14.86%
SD	10.01%	8.26%	18.74%	18.92%
Median	44.00%	20.00%	0.00%	8.00%
KW χ^2^		**25.628**		
*p*		**< 0.001**		
Mann-Whitney test (p-value)				
PTFE *vs.* PCD		< 0.001		
PTFE *vs.* PM		< 0.001		
PTFE *vs.* PMS		< 0.001		
PCD *vs.* PM		0.057		
PCD *vs.* PMS		0.331		
PM *vs*. PMS		0.352		

SD = standard deviation; PTFE: polytetrafluoroethylene expanded; PCD: polypropylene encapsulated with polydioxanone and coated with oxidized cellulose; PM: polypropylene mesh; PMS: polypropylene mesh coated with silicone.

**Table 5:** Frequency of hematoma/seroma per type of mesh.

Hematoma/seroma		Mesh				Total	chi-square	*p*	Fisher *p*
		PTFE	PCD	PM	PMS				
Yes	N	15	10	3	5	**33**			
	%	100.00%	66.70%	21.40%	35.70%	**56.90%**	21.960	< 0.001	< 0.001
No	N	0	5	11	9	**25**			
	%	0.00%	33.30%	78.60%	64.30%	**43.10%**			
Total	**N**	**15**	**15**	**14**	**14**	**58**			
	**%**	**100.00%**	**100.00%**	**100.00%**	**100.00%**	**100.00%**			

SD = standard deviation; PTFE: polytetrafluoroethylene expanded; PCD: polypropylene encapsulated with polydioxanone and coated with oxidized cellulose; PM: polypropylene mesh; PMS: polypropylene mesh coated with silicone.

The presence of visceral adhesions to the mesh was similar between groups (*p* = 0.029, Fisher test; *p* = 8.989, chi-squared test; data not shown). The presence of adhesion to the omentum was also similar between groups (*p* = 0.097, Fisher test; *p* = 6.074, chi-squared test).

The histological analysis revealed that the inflammation scores were significantly different between groups, as shown in Table 6. The investigation of COX2 by immunohistochemistry in the tissue between the skin and the mesh revealed significantly higher positivity for the ePTFE group (Tables 7 and 8). For the interface between the peritoneum and the mesh, there was also a significant difference between groups (Table 9).

**Table 6:** Inflammation scores per type of mesh.

Inflammation	PTFE	PCD	PM	PMS
N	15	15	14	14
Mean	20.93	20.2	18.64	18.21
SD	0.799	0.676	1.277	1.251
Media	21	20	19	18
KW χ^2^		**35.051**		
*p*		**< 0.001**		
Mann-Whitney test (*p*-value)				
PTFE *vs*. PCD		0.009		
PTFE *vs*. PM		< 0.001		
PTFE *vs*. PMS		< 0.001		
PCD *vs*. PM		0.001		
PCD *vs.* PMS		< 0.001		
PM *vs.* PMS		0.361		

SD = standard deviation; PTFE: polytetrafluoroethylene expanded; PCD: polypropylene encapsulated with polydioxanone and coated with oxidized cellulose; PM: polypropylene mesh; PMS: polypropylene mesh coated with silicone.

**Table 7:** Percentage of skin COX2 positivity in the interface between the skin and the mesh, per type of mesh.

COX2	PTFE	PCD	PM	PMS
N	12	14	14	13
Mean	2.17	1.21	1.14	1.31
SD	0.389	0.426	0.363	0.48
Median	2	1	1	1
KW χ^2^		**25.542**		
*p*		**< 0.001**		
Mann-Whitney Test				
PTFE *vs.* PCD	*p*		< 0.001	
PTFE *vs.* PM	*p*		< 0.001	
PTFE *vs.* PMS	*p*		< 0.001	
PCD *vs.* PM	*p*		0.628	
PCD *vs.* PMS	*p*		0.587	
PM *vs.* PMS	*p*		0.312	

SD = standard deviation; PTFE: polytetrafluoroethylene expanded; PCD: polypropylene encapsulated with polydioxanone and coated with oxidized cellulose; PM: polypropylene mesh; PMS: polypropylene mesh coated with silicone.

**Table 8:** COX2 positivity in the interface between the skin and the mesh, per type of mesh.

COX2	PTFE	PCD	PM	PMS
N	12	14	14	13
Mean	2.67	1.93	1.64	1.77
SD	0.492	0.267	0.497	0.599
Median	3	2	2	2
KW *χ* ^2^		**21.357**		
*p*		**< 0.001**		
Mann-Whitney Test				
PTFE *vs*. PCD	*p*		< 0.001	
PTFE *vs*. PM	*p*		< 0.001	
PTFE *vs*. PMS	*p*		0.001	
PCD *vs*. PM	*p*		0.07	
PCD *vs.* PMS	*p*		0.315	
PM *vs.* PMS	*p*		0.605	

SD = standard deviation; PTFE: polytetrafluoroethylene expanded; PCD: polypropylene encapsulated with polydioxanone and coated with oxidized cellulose; PM: polypropylene mesh; PMS: polypropylene mesh coated with silicone.

**Table 9:** COX2 positivity in the interface between the peritoneum and the mesh, per type of mesh.

COX2	PTFE	PCD	PM	PMS
N	12	14	14	13
Mean	1.75	1.36	1.14	1
SD	0.754	0.497	0.363	0
Median	2	1	1	1
KW *χ* ^2^		**13.059**		
*p*		**0.005**		
Mann-Whitney Test (*p*-value)				
PTFE *vs.* PCD		0.164		
PTFE *vs.* PM		0.017		
PTFE *vs.* PMS		0.002		
PCD *vs.* PM		0.199		
PCD *vs.* PMS		0.019		
PM *vs.* PMS		0.165		

SD = standard deviation; PTFE: polytetrafluoroethylene expanded; PCD: polypropylene encapsulated with polydioxanone and coated with oxidized cellulose; PM: polypropylene mesh; PMS: polypropylene mesh coated with silicone.

## Discussion

Although the correction of abdominal wall hernias with the mesh is one of the most frequent interventions in the daily life of the general surgeon, there are still many doubts about the host response to various types of mesh used in this procedure. Intraperitoneal mesh, sometimes with direct contact with abdominal organs, has been increasingly used^21^ and there are more than 600 products available^22^, most still pending clinical trials before use in humans. A gap in the literature, that this study tried to fill, was the study of inflammatory reactions, scarring and postoperative complications with different types of products, as adherences, intestinal fistulae and infection are common complications^23^ ^,^ ^24^. The idea of coating mesh with physical or chemical barriers to adherences was promising, but these substances could potentially impair incorporation and increase the risk of infection. In this study, the polypropylene mesh coated with silicone (PMS) had the best incorporation and the lowest area of adherences.

The higher rates of visceral adherences with the polypropylene mesh (PM) in this study was in accordance with some other studies^21^ ^,^ ^25^. Other authors^26^ found no significant differences in visceral adherences, suggesting that absorbable compounds that cover the mesh, with late degradation, could be the explanation. It was proposed that mesh covered with Goretex or expanded polytetrafluoroethylene (ePTFE) would create a “neoperitoneum”^27^, preventing the adherence to abdominal viscera^25^ ^,^ ^27^. However, the absorbable material could also increase the inflammatory process^26^ ^,^ ^28^, as in fact observed in our study, in which the ePTFE group presented higher incidence of collections along its surface (100%), while polypropylene mesh (PM) had the lowest (21%; p<0.001). Contradictory results in other studies^28^ ^,^ ^29^ may be due to differences in the experimental animals, mesh porosity and experimental or surgical methodology.

There are studies in the literature also in Wistar rats evaluating the intraperitoneal adherences that are seen with mesh covered with silicone. Takács *et al*.^30^ compared two different silicone mesh products and observed lower adherence with the laminar surgical silicon mesh. Baracs *et al*.^31^ obtained results that were similar to ours. In their study, they compared a silicone-covered mesh with polypropylene meshes and observed that the silicone-covered product significantly decreased the formations of adhesion. However, our study was more comprehensive: besides comparing the silicone with the polypropylene mesh, we also compared the abdominal adhesions with the ePTFE and the PCD mesh, which are products used in the intraperitoneal position. In this study, the PMS group presented the lowest adhesion area (63.1%) and grade 1 adhesion (22.4%) among all the evaluated products. The PCD group showed an intermediate area of adhesion and a looser adhesion (grade 2), and the ePTFE and PM groups presented almost the total area of adherence (99.8% and 97.7%, respectively). However, grade 2 adhesions predominated in the ePTFE group and grade 4 in the PM (86.7% and 64.3%). As the mesh in the PCD group contained absorbable material, it resulted in a greater inflammatory process and adhesions than in the ePTFE and PMS groups. We believe that the PMS group was the mesh that presented less intraperitoneal adhesion and between the viscera and the mesh due to the fact that it does not contain absorbable material and presents a long- lasting silicone barrier.

In our study, we observed that the group ePTFE had the higher percentage of area without incorporation (42%; p<0.001), while the PCD, PM e PMS groups had no significant difference between them (15.4%; 9.4%; e 14.8% respectively). In the study by Schreinemacher*et al*.^32^, three types of meshes were compared: polypropylene, polypropylene coated with carboxymethyl cellulose and polypropylene coated with ePTFE, without significant differences between them. In the experiment by Raptis*et al*.^24^, the ePTFE mesh was not well incorporated, since it became encapsulated when fixed intraperitoneally. As the incorporation of the meshes is related to fibroblasts and collagen infiltration in the material and it is directly associated with the inflammatory process caused by the product, we believe that, in the case of the ePTFE mesh, the presence of micropores hinders incorporation, since the mesh prevents the free passage and the infiltration of these components into its structure. The PMS group presented better incorporation than the ePTFE Group and no difference was observed in relation to the groups containing polypropylene. Possibly these findings occurred because the parietal face of the mesh in the PMS group has polypropylene in one of its surfaces, similar to the PCD and PM groups —and different from the ePTFE mesh which, although presenting a texture difference, is made of the same material on both surfaces.

No other investigators studied COX2 in hernia repair mesh products, and we have shown a higher inflammation score, evaluated by the COX2 analysis, with the ePTFE group in this study (p< 0.001) — while in the studies by Raptis*et al*.^24^ and Matthews *et al.* ^25^, the ePTFE had the lowest inflammatory reaction. COX2 is a proinflammatory substance that is the precursor to the arachidonic acid pathway, also involved with angiogenesis in inflammatory and neoplastic processes. These stable molecules are classically considered markers of inflammatory response^20^. There are no other similar investigations of COX2 by immunohistochemistry in mesh studies. In our group, Pereira-Lucena *et al*.^19^ investigated COX2 in a mesh fixed extraperitoneally, observing a higher concentration and percentage of this marker in the polypropylene mesh. In our results, the ePTFE group presented the highest inflammatory response, both by the scoring histological evaluation and in the COX2 immunohistochemistry evaluation, which confirms the greater inflammation caused by the mesh. There is still doubt as to whether this inflammatory response occurred due to the presence of the collection, or as a response of the mesh itself. This should be further investigated regarding a possible multifactorial cause for the inflammation: the presence of seroma (as mentioned by Raptis *et al.* ^24^), the size of pores, the mesh density and different materials used in coating. The comparisons should be made in studies in which there is an experimental suture fixing mesh to the abdominal wall, and not only intraperitoneal insertion of mesh products with no replacement of parietal tissues.

Deerenberg *et al.* ^33^ used the same mesh and same surgical procedure used in our study, but in a contaminated environment. The ePTFE had the worst result in that scenario, both in incorporation and infection rates, because the micropores probably facilitated the infiltration and proliferation of bacteria and impaired the action of defense cells. Besides, its visceral hydrophobic face would decrease tissue cell adhesion and allow the free passage of bacteria to the implant surface.

## Conclusions

Among all types of mesh studied, the polypropylene mesh (PM) had higher density of adherences, larger area of adherences, adherences to organs and percentage of incorporation. The polytetrafluoroethylene expanded mesh (ePTFE) had the higher area of adherences and lower incorporation. The polypropylene mesh coated with silicone (PMS) performed best in inflammation score, had a higher incorporation and lower area of adherences, and it was considered the best type of mesh regarding inflammation, adherences and incorporation. However, this is an experimental study with rats, with short follow-up, and there are no other studies that could be compared to ours in the literature yet.
